# Utility of Hybrid Endoscopy Combining Small‐Bowel Capsule Endoscopy and Conventional Ileocolonoscopy in Crohn's Disease

**DOI:** 10.1002/deo2.70295

**Published:** 2026-02-23

**Authors:** Takahiro Ito, Kohei Matsunaga, Yuya Ohno, Muchir Shi, Atsuo Maemoto

**Affiliations:** ^1^ Inflammatory Bowel Disease Center Sapporo Higashi Tokushukai Hospital Hokkaido Japan

**Keywords:** capsule endoscopy, Crohn's disease, endoscopic remission, ileocolonoscopy, treat‐to‐target

## Abstract

**Objectives:**

To evaluate the safety and utility of hybrid endoscopy for whole‐bowel assessment in Crohn's disease (CD) and its role in treatment decision‐making.

**Methods:**

This single‐center retrospective study included 125 patients with CD in clinical remission (Crohn's Disease Activity Index ≤150) who underwent hybrid endoscopy between February 2021 and September 2023. These patients were compared with 53 contemporaneous patients who underwent small‐bowel capsule endoscopy (SBCE) alone. Endoscopic remission (ER) was defined as Capsule Endoscopy Crohn's Disease Activity Index <3.5 and Simple Endoscopic Score for Crohn's Disease ≤2. Relapse rates were analyzed according to ER achievement and treatment modification using the Kaplan–Meier method.

**Results:**

Hybrid endoscopy resulted in a shorter small‐bowel transit time (184 min vs. 243 min, *p* < 0.01) but better bowel cleansing than SBCE alone. Complete small‐bowel visualization was achieved in 88.8% of patients following hybrid endoscopy, and 66 of 125 patients achieved ER. Patients without ER who did not undergo treatment intensification displayed significantly higher relapse rates (*p* = 0.028). Hybrid endoscopy was preferred by 82% of patients.

**Conclusions:**

This novel hybrid endoscopy approach permits a comprehensive assessment of CD activity using currently available technology. Hybrid endoscopy may be helpful in guiding treatment decisions, particularly treatment intensification in patients who have not achieved ER.

## 1 Introduction

Crohn's disease (CD) is a chronic inflammatory bowel disease characterized by transmural inflammation affecting any part of the gastrointestinal tract, although it most commonly involves the small bowel and colon. Despite achieving clinical remission, subclinical inflammation frequently persists and contributes to cumulative bowel damage, leading to complications including strictures and abscesses, ultimately necessitating surgical intervention. Early therapeutic intervention based on objective assessments of mucosal inflammation is essential to prevent disease progression and improve long‐term outcomes.

The treat‐to‐target strategy in CD emphasizes the importance of achieving endoscopic remission (ER) as a therapeutic goal beyond clinical symptom control [1]. Biomarkers, including C‐reactive protein (CRP), leucine‐rich alpha‐2 glycoprotein (LRG), and fecal calprotectin, assist in monitoring disease activity and serve as surrogate markers of intestinal inflammation [2]. Although fecal calprotectin has good correlation with endoscopic findings, particularly in colonic disease, its ability to detect small‐bowel inflammation shows greater variability [3]. Endoscopic evaluation remains the gold standard for assessing intestinal inflammation [4]. As CD affects both the small and large bowel, comprehensive endoscopic assessment of the entire disease extent is ideal for accurate treatment decision‐making.

Currently available endoscopic methods for evaluating small‐bowel CD include balloon‐assisted enteroscopy and small‐bowel capsule endoscopy (SBCE) [5, 6]. However, balloon‐assisted enteroscopy is technically demanding and time‐consuming, and it rarely achieves complete small‐bowel examination [6]. Although panenteric capsule endoscopy can evaluate both the small and large bowel, and it has displayed promise in CD assessment, it is not approved in Japan, and it carries technical challenges, including longer examination times and potential incomplete colonic evaluation attributable to inadequate preparation or rapid transit. SBCE effectively visualizes the small bowel, but colonoscopy is additionally required for large‐bowel assessment, increasing the patient burden through multiple procedures and bowel preparations.

To address these limitations and provide a practical solution using currently available technology, we developed an original “hybrid endoscopy” approach combining SBCE and conventional ileocolonoscopy (CS) performed on the same day to simultaneously evaluate the small and large bowel. This innovative strategy offers several advantages over panenteric capsule endoscopy: immediate availability in clinical practice, the ability to obtain tissue biopsies during colonoscopy, and more reliable visualization of colonic mucosa. This study compared the utility of hybrid endoscopy for pan‐intestinal assessment in CD in comparison to SBCE alone. To our knowledge, this is the first study to systematically evaluate the feasibility, safety, clinical utility, and impact of this hybrid endoscopy on treatment decision‐making in patients with CD in clinical remission.

## 2 Methods

### 2.1 Study Design and Patients

This single‐center retrospective study was conducted at Sapporo Higashi Tokushukai Hospital (Sapporo, Japan) between February 2021 and September 2023. We included consecutive patients with established CD in clinical remission (Crohn's Disease Activity Index [CDAI] ≤150) who underwent hybrid endoscopy combining SBCE and CS on the same day. Patients with a stoma were excluded from this study. All patients were diagnosed with CD based on the criteria of the Japanese Ministry of Health, Labor and Welfare. The patients’ clinical data, including demographics, disease history, and treatment responses, were collected from their medical records.

### 2.2 Hybrid Endoscopy Procedure

Patients underwent bowel preparation using standard ileocolonoscopy preparation regimens. Specifically, some patients took an oral bowel preparation at home before visiting the hospital to undergo SBCE (PillCam SB3, Medtronic, Dublin, Ireland) and CS. Conversely, some patients underwent SBCE after arriving at the hospital, after which they took an oral bowel preparation and underwent CS.

### 2.3 Comparison With Standard SBCE

To evaluate the impact of bowel preparation on SBCE performance, we compared patients who underwent hybrid endoscopy with 53 contemporaneous patients without intestinal stomas who underwent SBCE alone (without same‐day colonoscopy) at the same hospital and time period. The assessed parameters included gastric transit time, small‐bowel transit time, bowel cleansing quality, complete small‐bowel visualization rate, capsule retention, and adverse events. Cleansing was scored as poor (0), fair (1), good (2), or excellent (3).

### 2.4 Endoscopic Activity Assessment

Small‐bowel inflammation was assessed using the Capsule Endoscopy CDAI (CECDAI) [7], which separately scores inflammation (0–5), the extent of disease (0–3), and the presence of stricture (0–3) for the proximal and distal small‐bowel segments. CECDAI ≥3.5 indicates active disease, whereas CECDAI <3.5 denotes ER in the small bowel [8].

Large‐bowel and terminal ileum inflammation was evaluated using the Simple Endoscopic Score for CD (SES‐CD) [9], which assesses the ulcer size, ulcerated surface, affected surface, and presence of stricture across five segments (ileum, right colon, transverse colon, left colon, and rectum).

ER in hybrid endoscopy was defined as achieving CECDAI <3.5 and SES‐CD ≤2.

### 2.5 Treatment Interventions

Based on the hybrid endoscopy results, attending physicians made treatment decisions as follows: treatment intensification, which included (but was not limited to) dose escalation of biologics, switch to alternative biologics, addition of immunomodulators, and addition of corticosteroids; treatment de‐escalation, which involved reducing the dose of biological agents, extending the dosing interval, or discontinuing treatment; and no treatment modification. The decision to intensify treatment was primarily based on the absence of ER (CECDAI ≥3.5 or SES‐CD >2), with consideration of biomarker levels (elevated CRP or LRG), patient symptoms, and disease history.

### 2.6 Clinical Follow‐Up and Outcomes

The relapse rate following hybrid endoscopy was examined according to the achievement of ER and the use of treatment interventions. Clinical relapse was defined as worsening symptoms requiring a change in treatment. Patient acceptability of hybrid endoscopy was also investigated via a questionnaire.

### 2.7 Statistical Analysis

Continuous variables were presented as the median (range) and compared using the Mann–Whitney U test. Categorical variables were presented as numbers (percentages) and compared using Fisher's exact test. Relapse‐free survival was analyzed using the Kaplan–Meier method with the log‐rank test for group comparisons. Statistical significance was set at *p* < 0.05. All analyses were performed using EZR (Saitama Medical Center, Jichi Medical University, Saitama, Japan), a graphical user interface for R (The R Foundation for Statistical Computing, Vienna, Austria).

## 3 Results

### 3.1 Patient Characteristics

In total, 125 patients with CD in clinical remission underwent hybrid endoscopy during the study period. The baseline characteristics of the patients are summarized in Table 1. The median patient age was 33 years (range, 17–70), and most patients were male (78.4%). The median disease duration was 119 months (range, 11–464). The disease had an ileocolonic, isolated ileal, and isolated colonic location in 55.2%, 35.2%, and 9.6% of patients, respectively. The disease behavior was inflammatory, stricturing, and penetrating in 61.6%, 20.8%, and 17.6% of patients, respectively. In total, 29.6% of patients had undergone prior intestinal resection. At baseline, 80.8% of patients were receiving biologics, and 49.0% were on immunomodulators. Patency capsule examination was performed in 89 patients (71.2%) prior to SBCE to assess capsule passage safety, particularly in patients with stricturing disease or prior surgery.

**TABLE 1:** Baseline characteristics of the study patients.

Characteristics (*N* = 125)	*n* (%) or median (range)
Age	33 (17–70)
Male sex	98 (78.4)
Disease duration (months)	119 (11–464)
Disease location (ileum/colon/ileocolon)	44 (35.2)/12 (9.6)/69 (55.2)
Disease behavior (inflammatory/stricturing/penetrating)	77 (61.6)/26 (20.8)/22 (17.6)
History of bowel resection	37 (29.6)
Anal fistula	8 (6.4)
Patency capsule examination	89 (71.2)
Disease activity	
CDAI	38 (0–145)
Albumin (g/dL)	4.5 (3.6–5.4)
CRP (mg/dL)	0.05 (0.01–1.62)
LRG (*N* = 110, µg/mL)	11.1 (6.6–37.2)
Endoscopic activity	
CECDAI	1.0 (0–20)
SES‐CD	2.0 (0–21)
Medications	
Elemental diet (≥900 kcal/day)	36 (28.8)
Steroids (prednisolone or budesonide)	2 (1.6)
Immunomodulators	60 (49.0)
Biologics	101 (80.8)

### 3.2 Impact of Bowel Preparation on SBCE Performance

In the comparison between hybrid endoscopy (*n* = 125) and standard SBCE (*n* = 53), CDAI, CECDAI, and the gastric transit time were similar between groups (38 vs. 43, *p* = 0.24, 1 vs. 2, *p* = 0.37, 21 min vs. 26 min, *p* = 0.16, respectively; Table 2). However, the small‐bowel transit time was significantly shorter in the hybrid endoscopy group (184 min vs. 243 min, *p* < 0.01). Additionally, the median ileal cleansing score was significantly higher in the hybrid endoscopy group compared with the standard SBCE group (2 vs. 2, *p* < 0.01). Although the median values are identical, analysis using the Mann–Whitney U test reveals a significant difference. Moreover, the overall small‐bowel cleansing score (sum of the jejunal and ileal scores) was better in the hybrid endoscopy group (5 vs. 4, *p* = 0.02). Complete small‐bowel visualization was achieved in 88.8% (111/125) of patients who underwent hybrid endoscopy, versus 86.8% (46/53) of those who underwent standard SBCE (*p* = 0.80). In the 14 patients with incomplete small‐bowel visualization in the hybrid group, CECDAI was calculated based on the visualized segments; the median CECDAI was 3.5 (range 0–11), and 6 of these patients achieved ER by our criteria. Notably, all incomplete cases had visualization of the distal ileum, and the terminal ileum was assessed during ileocolonoscopy, minimizing impact on ER evaluation. All incomplete cases were included in the ER calculation and relapse analysis. No capsule retention or adverse events occurred in either group.

**TABLE 2:** Comparison of small‐bowel capsule endoscopy (SBCE) performance between hybrid endoscopy and standard SBCE.

	Hybrid endoscopy (*N* = 125)	Standard SBCE (*N* = 53)	*p*
CDAI, median	38	43	0.24^a^
CECDAI, median	1	2	0.37^a^
Gastric transit time (minutes), median	21	26	0.16^a^
Small‐bowel transit time (minutes), median	184	243	<0.01^a^
Jejunal cleansing score (0–3), median	3	2	0.48^a^
Ileal cleansing score (0–3), median	2	2	<0.01^a^
Overall small‐bowel cleansing score, median	5	4	0.02^a^
Complete small‐bowel visualization, *n* (%)	111 (88.8)	46 (86.8)	0.80^b^
Capsule retention, *n* (%)	0 (0)	0 (0)	—
Adverse events/device malfunction, *n* (%)	0 (0)	0 (0)	—

^a^ Mann–Whitney U test.

^b^ Fisher's exact test.

### 3.3 Endoscopic Activity and Treatment Interventions

**FIGURE 1:**
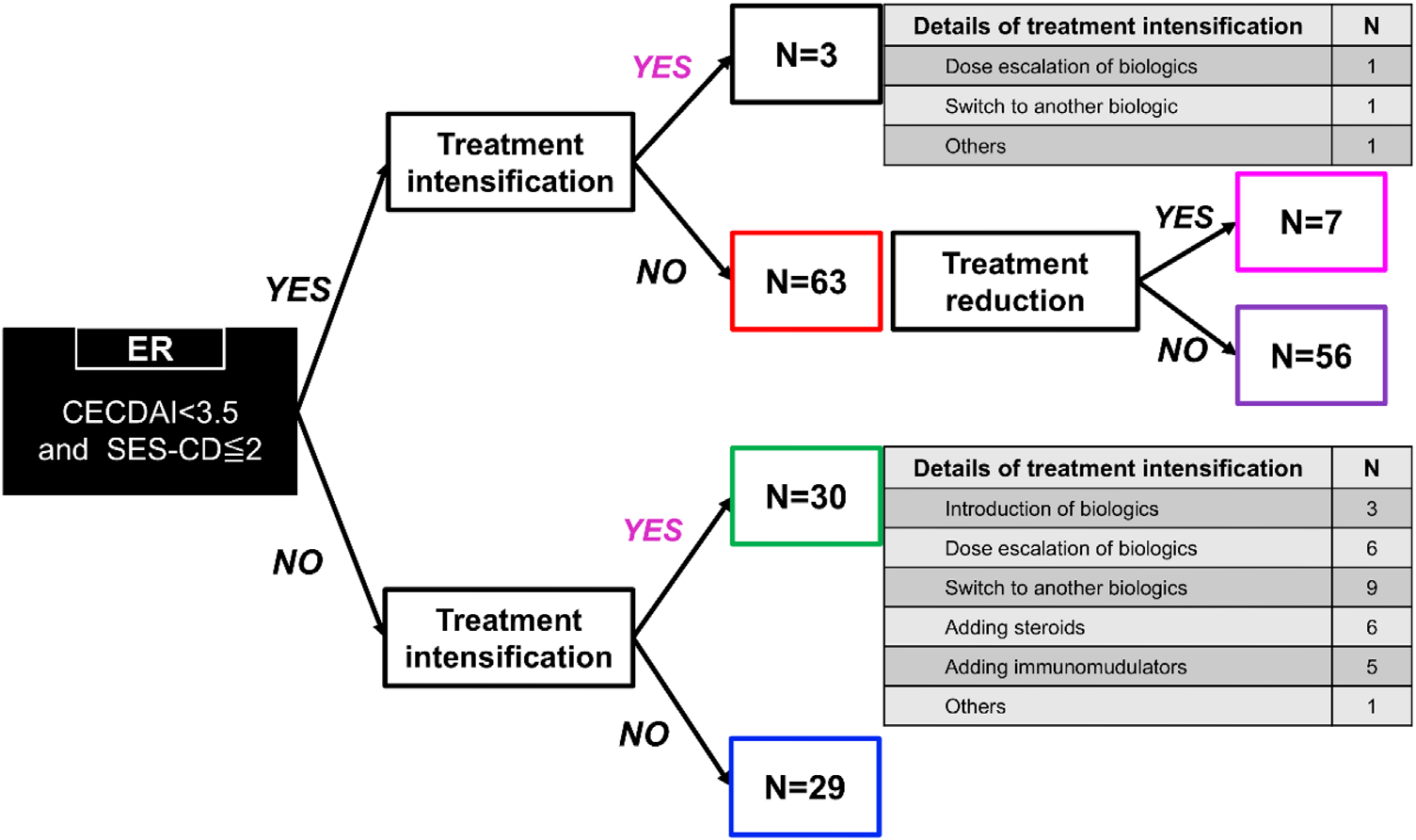
Endoscopic results and treatment intervention flowchart. The flowchart depicts the distribution of 125 patients with Crohn's disease (CD) in clinical remission according to the endoscopic remission (ER) status and treatment interventions following hybrid endoscopy. Details of the treatment intensification strategies are presented for each group.

Among 125 patients, 66 (52.8%) achieved ER (Figure 1). Of the 59 patients who did not achieve ER, 30 (50.8%) underwent treatment intensification including biologic introduction (*n* = 3), biologic dose escalation (*n* = 6), switch to an alternative biologic (*n* = 9), addition of corticosteroids (*n* = 6), addition of immunomodulators (*n* = 5), and others (*n* = 1). The remaining 29 patients did not undergo treatment intensification based on clinical judgment.

Among 66 patients achieving ER, three (4.5%) underwent treatment intensification (biologic dose escalation, *n* = 1; biologic switch, *n* = 1; other, *n* = 1), and seven (10.6%) underwent treatment de‐escalation. The remaining 56 patients continued their current therapy without modification.

### 3.4 Relapse Rates According to ER and Treatment Intervention

**FIGURE 2:**
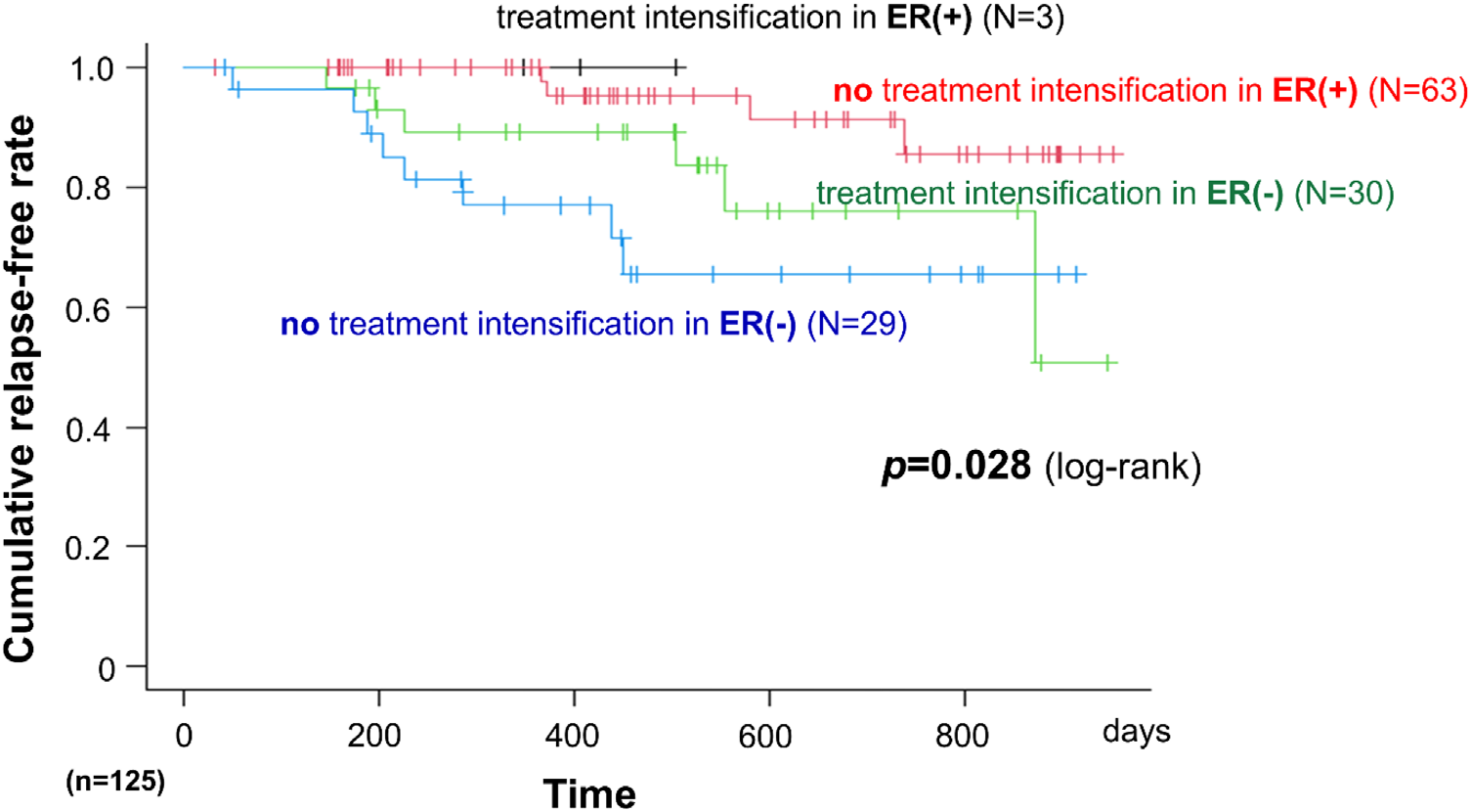
Kaplan–Meier analysis of relapse‐free survival according to endoscopic remission (ER) and treatment intensification. The Kaplan–Meier curves present the cumulative non‐relapse rates for 125 patients with Crohn's disease (CD) stratified by the ER and treatment intensification status. Four groups are compared: patients with ER who did not receive treatment intensification (red line, *N* = 63), patients with ER who received treatment intensification (black line, *N* = 3), patients without ER who received treatment intensification (green line, *N* = 30), and patients without ER who did not receive treatment intensification (blue line, *N* = 29).

Kaplan–Meier analysis demonstrated significant differences in relapse‐free survival among four groups stratified by ER achievement and treatment intensification (Figure 2, *p* = 0.028 by log‐rank test). Patients who did not achieve ER or undergo treatment intensification had the highest relapse rate. Conversely, patients who achieved ER maintained low relapse rates regardless of treatment intensification. Notably, patients who did not achieve ER who underwent treatment intensification displayed improved outcomes, with relapse rates intermediate between ER achievers and non‐intensified non‐ER patients.

### 3.5 Treatment De‐Escalation Following ER

**FIGURE 3:**
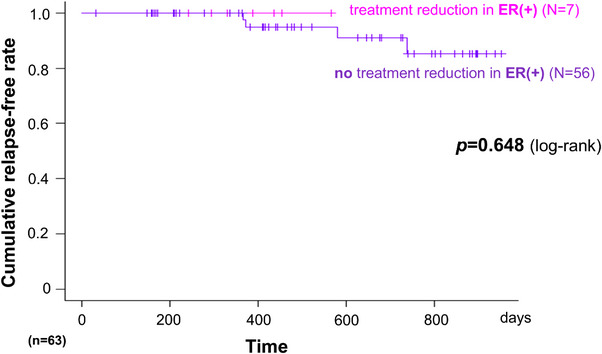
Kaplan–Meier analysis of relapse‐free survival according to treatment de‐escalation in endoscopic remission (ER)‐positive patients. The Kaplan–Meier curves compare cumulative non‐relapse rates between patients achieving ER who underwent treatment de‐escalation (red line, *N* = 7) and their counterparts who did not undergo treatment de‐escalation (black line, *N* = 56).

Seven patients underwent treatment de‐escalation following ER. Kaplan–Meier analysis showed no significant difference in relapse‐free survival between these patients and those who did not undergo de‐escalation (*p* = 0.648, Figure 3). However, given the very small sample size (*n* = 7), no conclusions can be drawn regarding the safety of treatment de‐escalation.

### 3.6 Patient Acceptability

**FIGURE 4:**
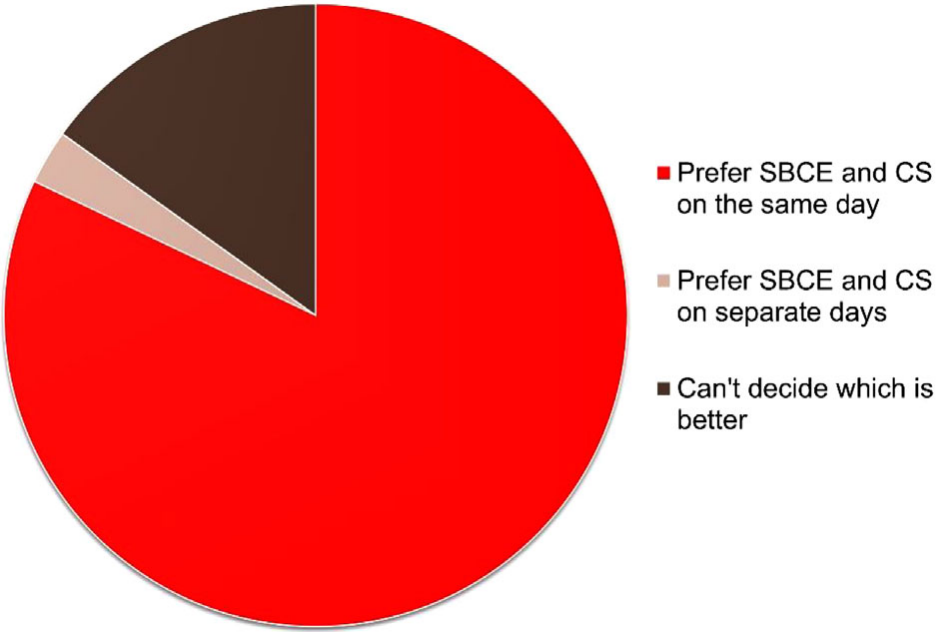
Patient acceptability of hybrid endoscopy. The pie chart presents patient preferences regarding the timing of small‐bowel capsule endoscopy (SBCE) and conventional ileocolonoscopy (CS) based on questionnaire responses (*N* = 106, response rate 84.8%).

Questionnaire responses were obtained from 106 of 125 patients (84.8%). The majority of respondents (82%) preferred undergoing SBCE and colonoscopy on the same day, whereas only 3% preferred to undergo the procedure on separate days (Figure 4). Additional questionnaire items revealed that 96% reported no or minimal embarrassment, 86% reported no or minimal fear, 99% reported no or minimal pain, and 84% found capsule swallowing easy. Compared with small bowel follow‐through, 87% found SBCE easier. The most common reasons for preferring same‐day procedures included single bowel preparation, a single hospital visit for work‐schedule convenience, and completing both examinations in one day.

### 3.7 Representative Cases

**FIGURE 5:**
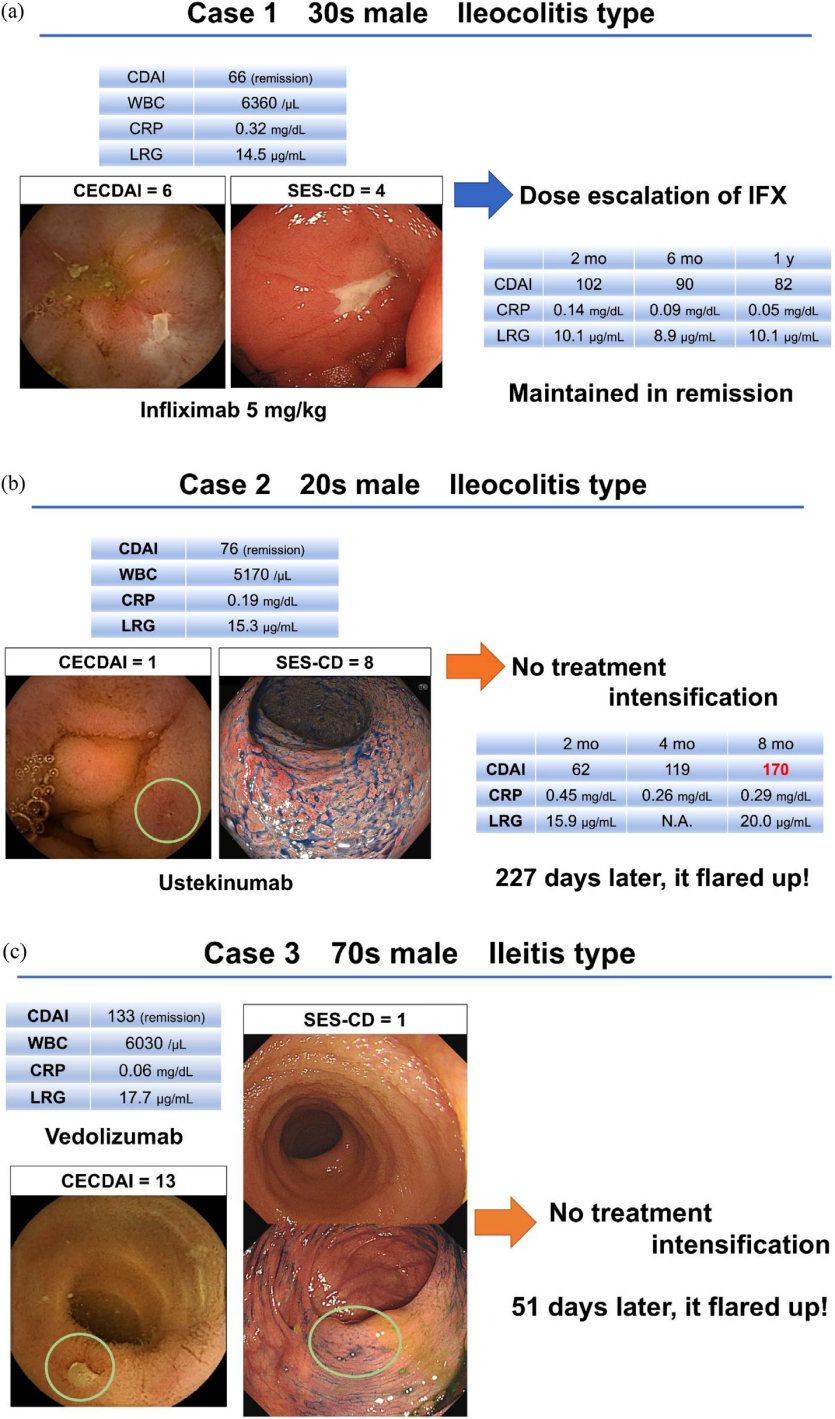
Representative cases. (a) A man in his 30s with ileocolonic Crohn's disease (CD) in clinical remission (Crohn's Disease Activity Index [CDAI] = 66) presented with a C‐reactive protein (CRP) level of 0.32 mg/dL and a leucine‐rich alpha‐2 glycoprotein (LRG) level of 14.5 µg/mL while receiving infliximab 5 mg/kg. Hybrid endoscopy revealed Capsule Endoscopy CDAI (CECDAI) = 6 and Simple Endoscopic Score for CD (SES‐CD) = 4. Following infliximab dose escalation, the patient maintained clinical remission at 2, 6, and 12 months. (b) A man in his 20s with ileocolonic CD in clinical remission (CDAI = 76) receiving ustekinumab exhibited minimal small‐bowel inflammation (CECDAI = 1) but significant colonic activity (SES‐CD = 8). Treatment intensification was not performed, and the patient experienced clinical relapse after 227 days. The areas outlined in pale green circles indicate active lesions. (c) A man in his 70s with ileal CD in clinical remission (CDAI = 133) receiving vedolizumab demonstrated minimal colonic inflammation (SES‐CD = 1) but significant small‐bowel activity (CECDAI = 13). Without treatment intensification, clinical relapse occurred rapidly at 51 days. The areas outlined in pale green circles indicate active lesions.

Figure 5 presents three cases in which hybrid endoscopy was performed in patients with CD in remission.

**Case 1** (Figure 5a) involved a man in his 30s with ileocolonic CD in clinical remission (CDAI = 66) receiving infliximab. Hybrid endoscopy revealed CECDAI of 6 and SES‐CD of 4, indicating no ER. Following dose escalation, he maintained remission with normalized biomarkers at 12 months.
**Case 2** (Figure 5b) involved a man in his 20s with ileocolonic CD in clinical remission (CDAI = 76) receiving ustekinumab, showing minimal small‐bowel inflammation (CECDAI = 1) but significant colonic activity (SES‐CD = 8). Without treatment intensification, clinical relapse occurred after 227 days.
**Case 3** (Figure 5c) involved a man in his 70s with ileal CD in clinical remission (CDAI = 133) receiving vedolizumab, showing minimal colonic inflammation (SES‐CD = 1) but significant small‐bowel activity (CECDAI = 13). Without treatment intensification, clinical relapse occurred after 51 days.

## 4 Discussion

This study highlighted the feasibility, safety, and clinical utility of our novel hybrid endoscopy approach combining SBCE and CS in the comprehensive assessment of CD activity. Our findings indicated that treatment decisions guided by hybrid endoscopy, particularly treatment intensification in patients without ER, can effectively prevent clinical relapse.

The concept of hybrid endoscopy addresses a significant gap in current endoscopic assessment strategies for CD. Although balloon‐assisted enteroscopy can theoretically evaluate both bowel segments, complete examination is rarely achieved [6]. Panenteric capsule endoscopy offers promise but remains unavailable in Japan and faces technical challenges [10]. By combining SBCE for small‐bowel assessment and colonoscopy for large‐bowel evaluation, hybrid endoscopy provides a practical solution for pan‐intestinal assessment that can be implemented immediately without requiring new device approvals, while permitting tissue sampling.

An important consideration for hybrid endoscopy is the potential impact of bowel preparation on SBCE performance. Our analysis demonstrated that bowel preparation shortened small‐bowel transit time while improving cleansing quality. Modern capsule systems incorporate adaptive frame rate technology that may compensate for faster transit; however, we lacked frame rate data to confirm this effect. The improved cleansing quality may have offset any disadvantage from faster transit.

The clinical utility of hybrid endoscopy was evidenced by its impact on treatment decisions and patient outcomes. In our cohort of patients in clinical remission, approximately half achieved ER using our strict criteria (CECDAI <3.5 and SES‐CD ≤2), highlighting the frequent dissociation between clinical and endoscopic disease activity. This finding aligns with the treat‐to‐target concept, emphasizing endoscopic healing as a key therapeutic goal.

Previous studies have demonstrated that capsule endoscopy findings can predict long‐term clinical outcomes in small bowel CD [11, 12]. Kaplan–Meier analysis revealed that relapse rates were highest in patients without ER who did not undergo treatment intensification, supporting treatment optimization based on objective endoscopic assessment.

Although seven patients underwent treatment de‐escalation following ER achievement without experiencing relapse during follow‐up, the sample size is too small to draw any conclusions regarding the safety of de‐escalation strategies. Future prospective studies with adequate sample sizes are required to evaluate whether hybrid endoscopy can guide treatment de‐escalation decisions.

The representative cases illustrated that significant inflammation in either bowel segment requires therapeutic attention, and partial disease assessment may lead to inadequate treatment.

Patient acceptability of hybrid endoscopy was high, likely reflecting reduced burden of same‐day procedures compared with separate examinations, including consolidated preparation and single‐day scheduling. Several limitations of this study warrant discussion. First, the single‐center retrospective design limited generalizability and introduced potential selection and information bias. The choice between hybrid endoscopy and standard SBCE was primarily determined by clinical indication for colonoscopy and logistical factors; patients requiring concurrent colonic evaluation were directed toward hybrid endoscopy. However, baseline disease activity (CDAI and CECDAI) showed no significant differences between groups, suggesting minimal selection bias based on disease severity. Second, endoscopic scoring was performed by treating physicians without a formal assessment of interobserver agreement, which might have influenced score reliability. However, both CECDAI and SES‐CD are validated scoring systems with established reproducibility when used by trained endoscopists. Third, treatment decisions were made by individual attending physicians rather than through a standardized protocol, introducing heterogeneity in management strategies. This pragmatic approach reflects real‐world clinical practice but limits the ability to draw definitive conclusions about optimal treatment algorithms. Fourth, our definition of ER used relatively strict criteria, and alternative thresholds might yield different results. Finally, our follow‐up period was relatively short, and longer‐term outcomes, including rates of surgery and long‐term complications, remain to be determined.

Despite these limitations, our study provides important evidence supporting the feasibility and clinical utility of hybrid endoscopy for CD assessment. Future prospective studies with standardized endoscopic evaluation, central reading of endoscopic images, protocolized treatment algorithms, and longer follow‐up will help establish optimal implementation strategies and confirm the clinical benefits suggested by our findings. Additionally, comparative studies with panenteric capsule endoscopy, when it becomes available in more regions, would be valuable to define the relative merits of each approach.

In conclusion, hybrid endoscopy combining SBCE and CS on the same day represents a novel, practical, and effective approach for comprehensive pan‐intestinal assessment of CD using currently available technology. This innovative strategy proved feasible and safe, and patient acceptance was high. Treatment decisions guided by hybrid endoscopy results, particularly treatment intensification in patients without ER, appear beneficial for preventing relapse. Hybrid endoscopy merits consideration as a valuable tool in the treat‐to‐target management strategy for CD, permitting immediate implementation potential without requiring new device approvals.

## Author Contributions

Takahiro Ito collected and analyzed the data and wrote the manuscript. Takahiro Ito, Kohei Matsunaga, Yuya Ohno, Muchir Shi, and Atsuo Maemoto performed capsule endoscopy and conventional colonoscopy. Atsuo Maemoto guided the writing of the manuscript and reviewed it. All authors have read and approved the final manuscript.

## Funding

The authors have nothing to report.

## Ethics Statement

The research protocol for this analysis was approved by the ethics committee of Tokushukai Group (Ref. no. TGE01388‐012) through the opt‐out method. The present study was conducted according to the guidelines stipulated in the Declaration of Helsinki, and the protocol adhered to the STROBE criteria for retrospective studies. The requirement for obtaining patient consent was not required owing to the retrospective nature of the study.

## Conflicts of Interest

The authors declare no conflicts of interest.

## Clinical Trial Registration

N/A

## Data Availability

Access to the data supporting the results of this study will be requested and reviewed with the principal investigator of this study through the corresponding author. The data are not available to the public due to privacy and ethical restrictions.
